# Pyriproxyfen-treated bed nets reduce reproductive fitness and longevity of pyrethroid-resistant *Anopheles gambiae* under laboratory and field conditions

**DOI:** 10.1186/s12936-021-03794-z

**Published:** 2021-06-22

**Authors:** Nelson Grisales, Rosemary S. Lees, James Maas, John C. Morgan, Dimitri W. Wangrawa, Wamdaogo M. Guelbeogo, Sagnon N’Fale, Steven W. Lindsay, Philip J. McCall, Hilary Ranson

**Affiliations:** 1 Department of Vector Biology, Liverpool School of Tropical Medicine, Liverpool, L3 5QA UK; 2 Present Address: World Mosquito Programme, Action On Poverty, Level 4, President Place, No. 93 Nguyen Du Street, District 1, Ho Chi Minh City, Vietnam; 3 Centre National de Recherche Et de Formation Sur Le Paludisme (CNRFP), Rue 1847 Avenue Kunda Yonré, 01 BP 2208 Ouagadougou, Burkina Faso; 4 Department of Biosciences, Durham University, Durham, DH1 3LE UK

**Keywords:** Pyriproxyfen (PPF), *Anopheles gambiae*, Insecticide-treated nets (ITNs), Juvenile hormone (JH), Olyset Duo®, Pyrethroid-resistance

## Abstract

**Background:**

The efficacy of insecticide-treated nets (ITNs) containing the insect growth regulator pyriproxyfen (PPF) and pyrethroid insecticides (PPF-ITNs) is being assessed in clinical trials to determine whether they provide greater protection from malaria than standard pyrethroid-treated ITNs in areas where mosquitoes are resistant to pyrethroids. Understanding the entomological mode of action of this new ITN class will aide interpretation of the results from these trials.

**Methods:**

*Anopheles gambiae *sensu lato (*s.l*.) mosquitoes from a susceptible laboratory strain were exposed to PPF-treated netting 24 h, 6 h, and immediately prior to, or 24 h post blood feeding, and the impact on fecundity, fertility and longevity recorded. Pyrethroid-resistant populations were exposed to nets containing permethrin and PPF (PPF-ITNs) in cone bioassays and daily mortality recorded. Mosquitoes were also collected from inside houses pre- and post-distribution of PPF-ITNs in a clinical trial conduced in Burkina Faso; female *An. gambiae s.l.* were then assessed for fecundity and fertility.

**Results:**

PPF exposure reduced the median adult lifespan of insecticide-susceptible mosquitoes by 4 to 5 days in all exposure times (p < 0.05) other than 6 h pre-blood meal and resulted in almost complete lifelong sterilization. The longevity of pyrethroid-resistant mosquitoes was also reduced by at least 5 days after exposure to PPF-ITNs compared to untreated nets, but was unaffected by exposure to standard pyrethroid only ITNs. A total of 386 blood-fed or gravid *An. gambiae s.l*. females were collected from five villages between 1 and 12 months before distribution of PPF-ITNs. Of these mosquitoes, 75% laid eggs and the remaining 25% appeared to have normal ovaries upon dissection. In contrast, only 8.6% of the 631 blood-fed or gravid *An. gambiae s.l.* collected post PPF-ITN distribution successfully oviposited; 276 (43.7%) did not oviposit but had apparently normal ovaries upon dissection, and 301 (47.7%) did not oviposit and had abnormal eggs upon dissection. Egg numbers were also significantly lower (average of 138/female prior distribution *vs* 85 post distribution, p < 0.05).

**Conclusion:**

Exposure to a mixture of PPF and pyrethroids on netting shortens the lifespan of mosquitoes and reduces reproductive output. Sterilization of vectors lasted at least one year under operational conditions. These findings suggest a longer effective lifespan of PPF-pyrethroid nets than reported previously.

**Supplementary Information:**

The online version contains supplementary material available at 10.1186/s12936-021-03794-z.

## Background

As pyrethroid resistance becomes more widespread among African malaria vectors existing tools reliant on this insecticide class, such as insecticide-treated nets (ITNs), are losing efficacy, and may be contributing to a recent rebound in malaria cases [1]. There is, therefore, a need for insecticides with novel modes of action for use in malaria vector control tools including ITNs [2].

Methoprene and pyriproxyfen (PPF) are insect growth regulators that mimic the action of the insect juvenile hormone (JH), which is essential for normal physiological development and maturation of juvenile insects. In holometabolous insects like mosquitoes it is essential for larval development and its downregulation is critical for metamorphosis and adult emergence [3, 4]. In anautogenous mosquito females oocyte development enters a ‘resting stage’ under the influence of JH [5] and its synthesis stops shortly after the blood meal and fat bodies and ovaries become receptive to the hormone 20-hydroxyecdysone which promotes egg development. Hence raising levels of JH, or JH analogues/mimics such as PPF, at this stage will disrupt oogenesis, potentially providing an effective means of mass sterilization by the auto-dissemination mechanism, reducing the size of future vector populations [7].

*Anopheles* mosquitoes exposed to PPF-treated net samples show reduced fertility, longevity and lifetime fecundity compared to unexposed controls [8, 9]. Combining PPF with pyrethroids in ITNs is one approach currently under evaluation to reduce transmission of malaria by pyrethroid-resistant anopheline mosquitoes; mosquitoes able to withstand exposure to the pyrethroid insecticide are expected to be sterilized, and potentially have shorter lifespans due to exposure to the insect growth regulator on the nets. The ability of these dual action nets to sterilize field populations has been demonstrated in experimental hut trials using PPF-ITNs from two manufacturers: Olyset Duo^®^ from Sumitomo Chemical Ltd (permethrin and PPF) and Royal Guard^®^ from Disease Control Technologies (alphacypermethrin and PPF). For both net types, mosquitoes collected from huts with PPF-ITNs had reduced fertility and fecundity compared to mosquitoes from huts containing pyrethroid only ITNs [10–12]. The potential of PPF-ITNs to reduce malaria transmission has been shown in a cluster randomized clinical trial conducted between 2014 and 2015, in an area of intense malaria transmission in Burkina Faso where vectors are highly resistant to pyrethroids. The entomological inoculation rate in clusters receiving Olyset Duo nets was approximately half that in control arms, containing pyrethroid only nets (rate ratio 0.49, 95% CI 0.32–0.66; p < 0.0001) and this resulted in a reduction in clinical malaria in children (incidence rate ratio 0·88 [95% CI 0.77–0.99; p = 0·04]).﻿ Epidemiological trials of Royal Guard nets are ongoing in Tanzania and Benin [13, 14].

To better understand the mode of action of PPF-ITNs, results are reported here from laboratory bioassays measuring the impact of PPF on the reproductive output and longevity of pyrethroid susceptible populations, and data on the impact of PPF-ITNs on survival and longevity of pyrethroid resistant populations from the laboratory and field. The study also sought to understand more about the biological efficacy of PPF-ITNs under field settings by measuring the fertility of mosquitoes collected from houses in the Burkina Faso clinical trial pre and post PPF-ITN distribution.

## Methods

### Mosquito strains

The Tiassalé 13 strain is a hybrid of *Anopheles gambiae *sensu stricto (*s.s*.) and *Anopheles coluzzii* which was collected in Côte d’Ivoire in 2013 since when it has been maintained in the insectaries of the Liverpool Insect Testing Establishment at the Liverpool School of Tropical Medicine under conditions described previously [15]. This strain is highly resistant to DDT, permethrin, deltamethrin, bendiocarb, dieldrin, partially susceptible to fenitrothion and regularly subjected to selection pressure with deltamethrin to maintain pyrethroid resistance. Hereafter this strain is referred to as the pyrethroid-resistant strain.

The Kisumu strain used was *An. gambiae s.s*. colony susceptible to all insecticides, originally collected in Kisumu, and obtained from the MR4 collections [16]. Hereafter this strain is referred to as the pyrethroid-susceptible strain. The strain was maintained in the insectary of the Centre National de Recherche et de Formation sur le Paludisme (CNRFP) in Ouagadougou, Burkina Faso, held at temperatures of 27–30 °C and relative humidity of 75%-95% with a 12 h light:12 h dark photoperiod. Larvae were fed TetraMin Baby^®^ fish food, and adults provided with 10% sucrose solution. Blood meals were provided by placing an immobilized rabbit on top of the cage, using different animals to feed treatment and control groups to avoid contamination.

Adults were reared from larvae collected from aquatic habitats in the villages of Naniagara, Tiefora and Bakaridjan in the Cascades District of south-western Burkina Faso and maintained in the CNRFP insectaries in Banfora, Burkina Faso to produce adult female F_0_ progeny for bioassays (coordinates of larval collection sites are provided in Additional file 1: A1). Larval collections were performed in water bodies around each village, including semi-permanent and temporary water bodies, between June 2013 and October 2015. *Anopheles* larvae of all stages were collected using hand dippers and transported to the insectaries in Banfora where they were reared as described above. Blood-fed females collected inside houses in the village of Naniagara were allowed to oviposit and their progeny reared to adults under the same conditions in the Banfora insectaries. Results from insecticide susceptibility tests with permethrin are reported in full elsewhere [17], but can be summarized as mortality rates of less than 20% in discriminating dose assays for mosquitoes from all three villages. Additional file 1: Table A2.

### The effect of relative time of exposure to pyriproxyfen-treated netting and blood feeding on insecticide-susceptible *Anopheles gambiae*

#### Exposing and blood feeding mosquitoes

To evaluate if the relative timing of contact with the PPF-treated net and the provision of a blood meal had any effect on PPF efficacy, mosquitoes from Kisumu strain were exposed to the net and blood-fed according to different regimes. A 1% w/w PPF-treated net and an untreated net with the same material and denier was provided by Sumitomo Chemical Co., Ltd. (Tokyo, Japan), and designed to have a release rate of PPF as close as possible to that of the Olyset Duo^®^ net. Mosquitoes were exposed to the net using a custom Deli pot bioassay: 25 ml clear plastic pots (height 28 mm, top diameter 50 mm base diameter of 40 mm) were prepared by cutting a large hole in the lid of the pot and a smaller (approximately 1 cm diameter) hole in the bottom (Additional file 2: Fig. S1). The lid and pot were assembled with a piece of either untreated or PPF-treated net between them. Groups of 10 mosquitoes were introduced by manual aspirator to the assembled plastic pot and exposed to untreated or PPF netting for three minutes.

To investigate the effect of time of PPF exposure relative to that of a blood meal, susceptible adult female mosquitoes were exposed to an untreated or PPF-treated net at 24 h, 6 h, and immediately (within 15 min) prior to, or 24 h post blood feeding. The 24-h pre-exposure treatment was to simulate mosquitoes exposed while trying unsuccessfully to blood-feed on a human host protected by a bed net and then succeeding on the following night. The 6 h pre-exposure treatment simulated an initial contact with a net followed by a successful feed on the same night. The 24 h post blood-feeding treatment represented mosquitoes that successfully took a blood meal and then were exposed to a net the following night. Each treatment group was matched with a negative control group exposed to an untreated net. Three-day old female mosquitoes were used for all experiments, except in the ‘24 h prior cohort’ where exposure occurred on day 3, and the blood meal offered on day 4.

#### Measuring mosquito longevity and lifelong fecundity

Kisumu strain mosquitoes from a single cohort were used for this experiment, exposed as described above in pools of 10 mosquitoes with at least 10 replicates per treatment. After exposure to the net, groups of mosquitoes from replicate exposures were pooled by treatment, into a 30 × 30 × 30 cm cage. Mosquitoes that were unable to stand or were dead were removed. A 10% sucrose solution was provided *ad libitum*. Individual mosquito mortality was recorded daily and dead mosquitoes were removed from the cages.

A blood meal was offered to each cage every week until all mosquitoes died. The number of fully engorged mosquitoes was observed immediately post blood-feeding, but all females were retained in the cage. Two days after each blood meal, a plastic dish with a filter paper, partially submerged in distilled water, was introduced in each cage to encourage oviposition. Mosquitoes were allowed to lay eggs for three days, before the paper was removed and the eggs counted using a dissection microscope. Temperature and relative humidity were recorded daily.

#### Measuring the fecundity, fertility and offspring viability of individual mosquitoes

In this experiment, six replicate groups of 10 mosquitoes from Kisumu strain were exposed per treatment described above, using a single cohort of mosquitoes. After exposure, mosquitoes were individually transferred to flat-bottomed 50 ml plastic cell culture tubes, each containing a piece of filter paper over a wet piece of cotton for oviposition and sealed with a piece of mesh held by a rubber band to provide air. A piece of cotton soaked in 10% sucrose solution was put on the mesh on top of the tubes. Individual oviposition was recorded daily for five days post blood meal. After five days, all remaining live mosquitoes which failed to oviposit were dissected and their ovaries scored for follicular development using observation under a microscope. The ovaries were visually scored as comprising either normal or abnormal (with no follicular growth or yolk deposition). Dead mosquitoes were discarded.

Individual egg batches were placed in separate disposable plastic pots (height 42 mm, top diameter 115 mm, base diameter 85 mm) with approximately 50 ml of distilled water. A pinch of ground TetraMin fish food was added to the pots daily after larval hatching. The total number of 2nd instar larvae produced and the number of adults emerging were recorded as estimated measurements of hatching ratio and adult production ratio, respectively, for every treatment batch.

#### Blood feeding mosquitoes through the pyriproxyfen-treated netting

To simulate those mosquitoes which successfully feed on a human host through a PPF-treated net, a 15 × 15 cm piece of the cage top was replaced with a piece of PPF-treated net through which a blood meal was offered to around 100 adult female susceptible mosquitoes (Kisumu strain). A negative control treatment consisted of a parallel cage with mosquitoes fed through the untreated mesh top of cages. Mosquitoes were left to feed freely for up to 20 min to allow complete blood meals to be taken, therefore, the precise length of exposure to the net was unknown.

Two experiments were performed in parallel to those described above using a subset of the same cohorts of mosquitoes. In the first experiment, longevity and lifelong fecundity was measured in two pools of mosquitoes, one allowed to feed through a PPF-treated net and one through an untreated net. In the second experiment, mosquitoes were isolated to measure individual fertility, fecundity, and offspring viability, as described above.

Measuring the impact on 24 h mortality and adult longevity of exposure to pyriproxyfen and/or pyrethroids in pyrethroid-resistant *An. gambiae *sensu lato (*s.l*.) colonies and field collected mosquitoes.

Pyrethroid-resistant mosquitoes from the Tiassalé 13 strain were tested in LSTM laboratories against pyrethroid-treated netting (Olyset^®^ Nets, 2% w/w permethrin, equivalent to 800 mg/m^2^ of finished net), PPF-treated netting (1% w/w PPF, equivalent to 400 mg/m^2^) and PPF ITNs (Olyset Duo^®^ nets, 2% permethrin, 1% PPF, equivalent to 800 and 400 mg/m^2^). Naniagara F_0_ adults reared from larval collections were tested in Banfora insectaries using PPF-ITNs and control nets only. Mosquitoes obtained from larval collections at Tiefora and Bakaridjan, between July and September 2014, were exposed to control nets, ITNs and PPF-ITNs.

Three to five day-old female adults were exposed to net samples in a WHO cone bioassay following the standard protocol [18] with the following modifications; first panels were not selected systematically from each side of the net, as suggested by the guidelines and second, 10 mosquitoes rather than the suggested five, were tested for each cone. Mosquitoes exposed in this manner were used to measure the immediate mortality effects and longer term impact on survival (Tiassalé and Naniagara only).

To measure the immediate effect of PPF-ITN exposure on mortality, mosquitoes were exposed to the nets for 3 min, with knockdown and mortality being recorded at 1 h and 24 h post-exposure. Untreated nets were used as negative controls. Mosquitoes were then offered a blood meal 24 h post net exposure. Unfed mosquitoes were discarded and engorged mosquitoes pooled in empty polyethylene buckets (85 oz) covered by a fine mesh. Mortality was recorded daily until all mosquitoes died. A piece of cotton containing 10% sucrose solution was available ad libitum and a blood meal offered weekly using either a Hemotek Membrane Feeding System (Hemotek Ltd., Blackburn, UK) with human blood procured from the non-clinical product stock from the UK blood bank, or by arm feeding in the case of Naniagara mosquitoes. In each net-treatment group, mortality comparisons were made with negative control nets (untreated nets).

Up to 10 replicate exposures of 10 individuals were performed per treatment for each experiment, although, as not all mosquitoes bloodfed, starting numbers were lower for some controls. Without the considerable resources needed to produce the high number of mosquitoes for this experiment, the cone bioassay experiments were not conducted for all nets simultaneously, but as paired tests comparing a treated net with an untreated control.

### Determination of the impact of implementation of pyriproxyfen ITNs on the reproductive output of *Anopheles gambiae s.l.* in Banfora district, Burkina Faso

#### Distribution of Olyset Duo nets and mosquito collection protocols

Five villages in the Cascades region of Burkina Faso were selected where ITNs (Olyset Nets^®^) were originally distributed to all households in May—June 2014 and replaced by PPF-ITNs (Olyset Duo^®^) from June 2014 to September 2015 in a stepped-wedge experimental design [19]. Mosquito collections were performed before and after replacement of ITNs with PPF-ITNs in five villages: Naniagara (Olyset Duo^®^ replacement in September 2014, mosquito collection in June 2014 and September 2015), Bakaridjan (Olyset Duo replacement in July 2015, mosquito collection in September 2014 and September 2015), Pont Maurice, Djomale and Sikané (Olyset Duo^®^ replacement in August 2015, mosquito collection in June and October 2015) (see Additional file 1: Table A1 for coordinates of villages).

Female blood-fed anopheline mosquitoes were collected inside houses in each of the study villages. The number of houses visited depended on the density of adult female *Anopheles* and ranged from 23 (Sikané) to 140 (Pont Maurice). For the ‘prior’ collections, i.e. collections done when only ITNs were present, mosquitoes were captured in every house that collectors were allowed to enter. For the post-replacement collections, mosquitoes were only collected in houses where PPF-ITNs were present. Collections started at 06.00 h and residents requested to keep windows and doors closed until mosquito collections were completed. Mosquitoes were collected indoors using Prokopack aspirators [20], transferred gently into mosquito cages and transported to the Banfora insectaries. Collections continued until approximately 100 females had been assessed for oogenesis, per village, per collection period.

#### Measurement of individual mosquito fecundity, fertility and offspring viability of wild mosquitoes

Individual blood-fed mosquitoes collected in Naniagara, Bakaridjan, Pont Maurice, Djomale and Sikané villages were transferred to flat bottomed 50 ml plastic cell culture tubes to measure oviposition rates and individual fecundity and fertility as described above. As mosquito size and/or species could be confounding factors when measuring reproductive outputs, a random sub-sample of individuals, collected post Olyset Duo distribution that either: a) laid healthy eggs, b) did not lay eggs but had normal ovary appearance on dissection, and c) did not lay eggs and had abnormal ovary appearance were taken for wing length measurement and species identification (determined following the SINE protocol.

### Statistical analysis

All statistical analyses were carried out using the R statistics package, v 4.02 [22]. Kaplan–Meier survival analyses were done using the survival [23] and survminor [24] packages and graphs drawn using the ggplot2 package [25]. Fisher Exact Probability Test was used to compare proportions of mosquitoes blood-fed or ovipositing in the experiments performed in the laboratory. Wing length analysis was done using the t.test function in R to produce mean and 95% confidence interval values.

## Results

### The effect of relative time of exposure to pyriproxyfen-treated netting and blood feeding on insecticide-susceptible *Anopheles gambiae*

#### Pyriproxyfen exposure reduces longevity in pyrethroid susceptible mosquitoes

**Fig. 1:**
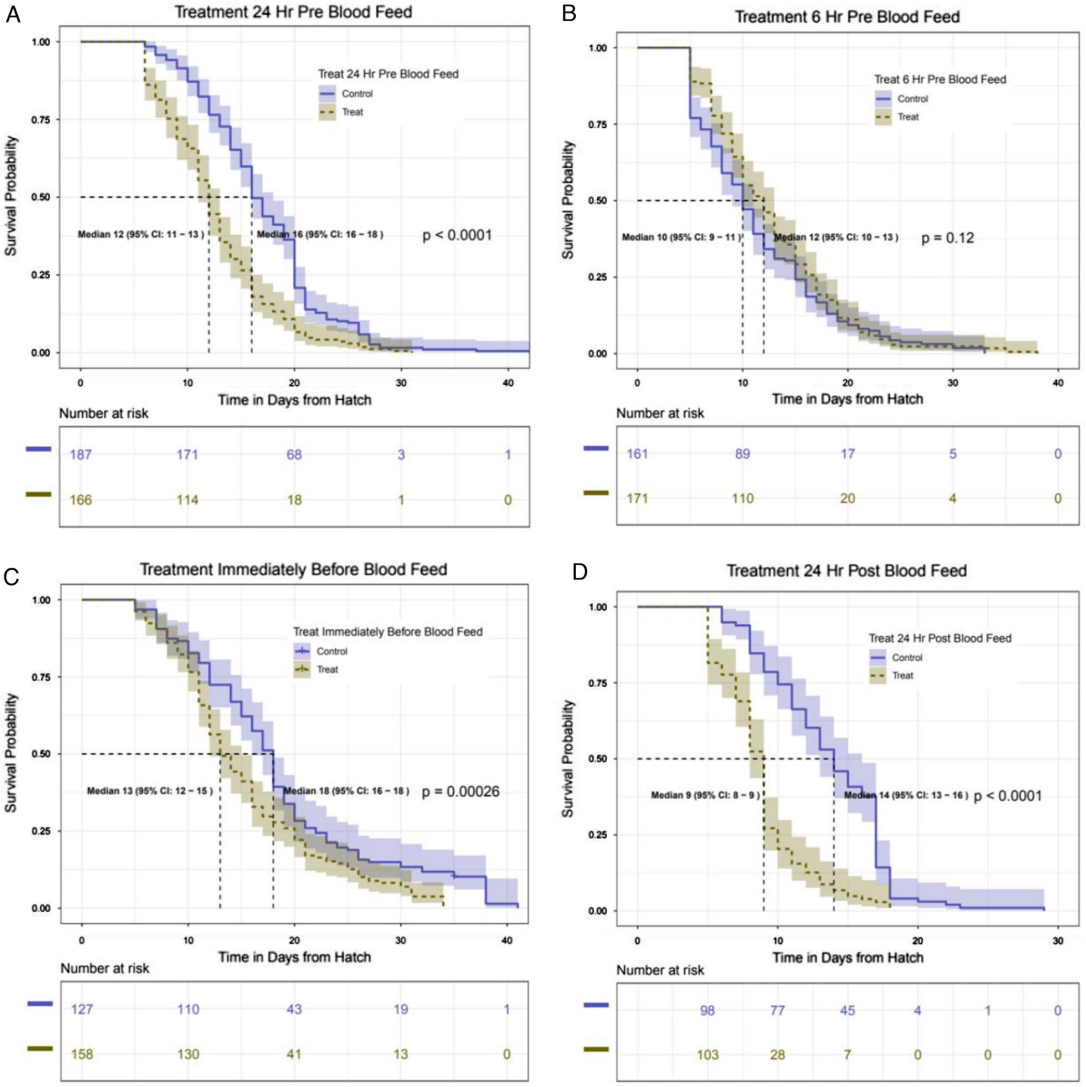
Survival curves for *An. gambiae s.s.* Kisumu strain mosquitoes exposed to PPF at different times relative to being offered a blood meal. Daily survival of insecticide susceptible mosquitoes after exposure to 1% PPF-treated netting (brown) or untreated netting (blue) 24 h (**A**), 6 h (**B**) or immediately before (**C**), or 24 h after a blood meal was offered (**D**). Mosquitoes were exposed to netting 4 days post-emergence

Kaplan–Meier survival curves for each of the groups of insecticide susceptible mosquitoes exposed to PPF-treated nets or untreated nets are shown in Fig. 1. In all cases, except the 6 h pre-blood meal treatment, exposure to the PPF-treated net increased the rate of mortality of the mosquitoes.

#### Pyriproxyfen exposure reduces lifelong fecundity in pyrethroid susceptible mosquitoes

Exposure to PPF-treated netting resulted in few eggs being laid (0–5 eggs/test), regardless of the timing of exposure in relation to the first blood meal (Table 1). Blood feeding rates (55%–100%) were similar between PPF-exposed and those exposed to untreated netting (p > 0.1).

**Table 1:** Blood feeding rates and egg production in different treatment groups in mosquitoes from Kisumu strain. The number of female mosquitoes that blood-fed, and the number of eggs they produced from successive opportunities to blood feed

Time blood meal was offered relative to exposure	Netting type	1st Blood meal		2nd Blood meal		3rd Blood meal		4th Blood meal		5th Blood meal	
		Blood-fed (n/N), (%)	Eggs Laid (N)	Blood-fed (N)	Eggs laid (N)	Blood-fed (N)	Eggs laid (N)	Blood-fed (N)	Eggs laid (N)	Blood-fed (N)	Eggs laid (N)
24 h prior	Untreated	149 / 187 (80)	6,551	127	5,215	12	160	7	216	3	122
	PPF-treated	125 / 167 (74)	0	55	0	1	0	1	0	0	0
6 h prior	Untreated	102 / 162 (62)	1,645	37	1,447	16	226	4	199	0	0
	PPF-treated	95 / 171 (55)	0	64	5	14	0	1	4	0	0
Immediately prior	Untreated	ND / 126	5,241	104	3,782	38	1,175	23	384	16	0
	PPF-treated	ND / 158	0	107	0	47	63	23	32	0	0
24 h post	Untreated	98 / 98 (100)	3,534	47	1,888	0	0	0	0	0	0
	PPF-treated	100 / 100 (100)	0	53	0	0	0	0	0	0	0

Control mosquitoes were exposed to untreated nets, and PPF treated were exposed to PPF-treated nets at 24 h prior, 6 h prior, immediately before, or 24 h post the first blood feeding opportunity, respectively. The number of mosquitoes offered a blood meal (Total) was recorded at the first blood feed, and the number of females that took a blood meal were recorded at each blood feeding. An error in recording resulted in missing data for the immediately prior cohort (ND = not determined)

#### Pyriproxyfen exposure reduces the fecundity, fertility and offspring viability of individual mosquitoes

**Fig. 2:**
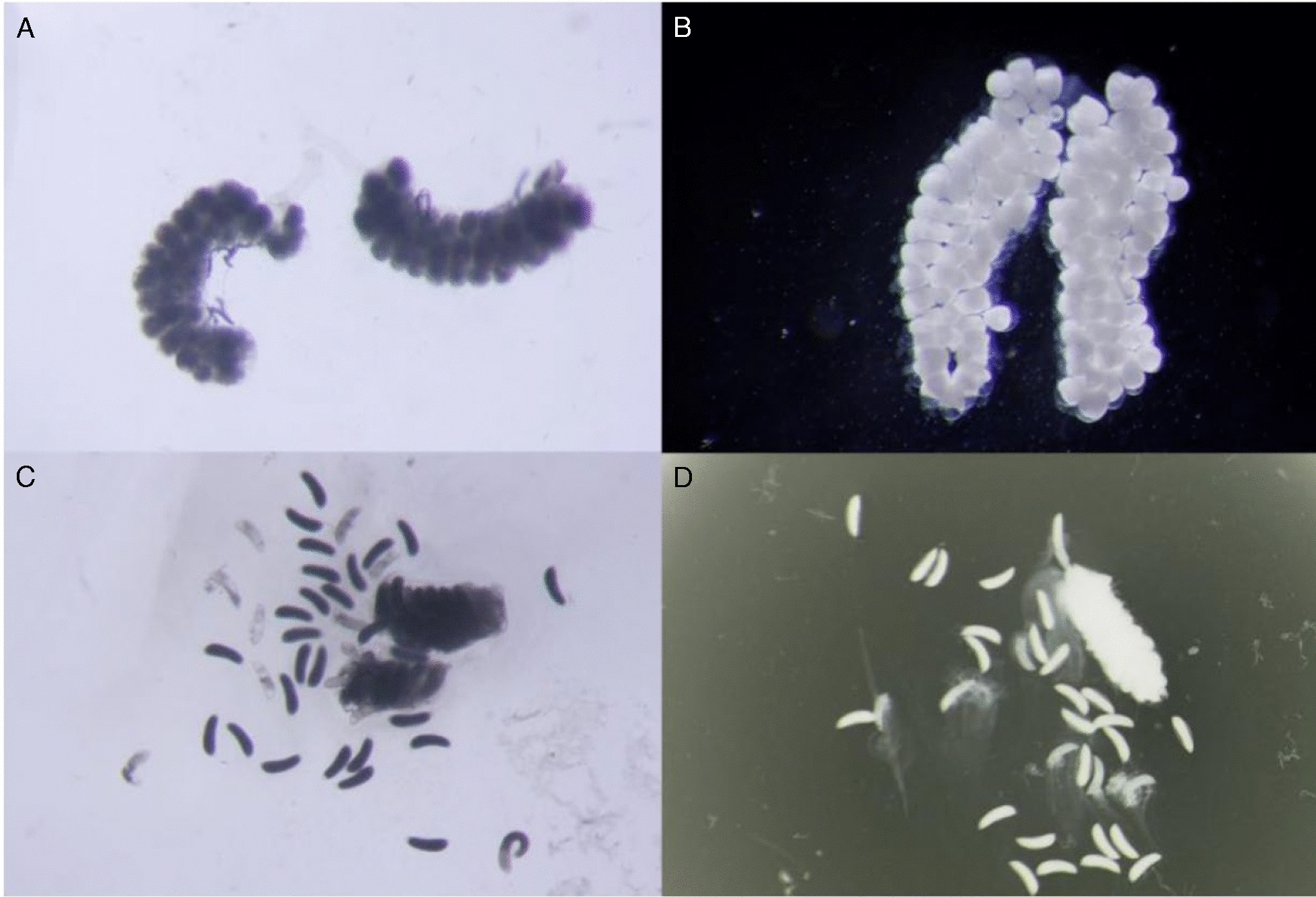
Morphology of eggs retained in ovaries of PPF exposed females compared to those in unexposed controls. Images are representative and were taken 4–5 days post exposure. **A** and **B**: Ovaries of mosquitoes exposed to PPF, showing round, non-detachable eggs. **C** and **D** Ovaries of unexposed females, showing normal, oval-shaped mature eggs. Scale: all images at 200X magnification (approximate)

In experiments where mosquitoes were able to oviposit separately, the oviposition rate in the groups exposed to untreated netting ranged from 76 to 88% but none of the mosquitoes exposed to PPF laid any eggs (Table 2). The morphology of the primary follicles was assessed in individuals which did not oviposit (Fig. 2). In the control group, 3% (1/35) had abnormal ovaries, compared to 94% (119/126) of mosquitoes exposed to PPF (p < 0.01). Larval hatch rates and adult emergence were recorded, but, as none of the treatment group laid any eggs, these results are not presented here.

**Table 2:** Number of eggs oviposited per individual female from Kisumu strain after a single blood feeding opportunity in mosquitoes exposed to a pyriproxyfen treated net at different time points

Time blood meal was offered relative to exposure	Netting type	Blood -fed (N)	No. (%) ovipositing	Mean no. eggs/mosquito	95% Confidence Interval
24 h prior	Untreated	41	36 (88%)	78.5	64.6–92.4
	PPF-treated	19	0 (0%)	0	0
6 h prior	Untreated	42	34 (81%)	88.4	75.3–101.6
	PPF-treated	26	0 (0%)	0	0
Immediately prior	Untreated	46	35 (76%)	58.1	39.7–76.5
	PPF-treated	36	0 (0%)	0	0
24 h post	Untreated	59	48 (81%)	69.7	57.6– 81.9
	PPF-treated	59	0 (0%)	0	0

Mosquitoes were exposed to untreated netting or PPF-treated netting at 24 h or 6 h before, or 24 h after a blood meal was offered

#### Blood feeding mosquitoes through pyriproxyfen-treated netting reduces longevity and lifelong fecundity in pyrethroid susceptible mosquitoes

**Fig. 3:**
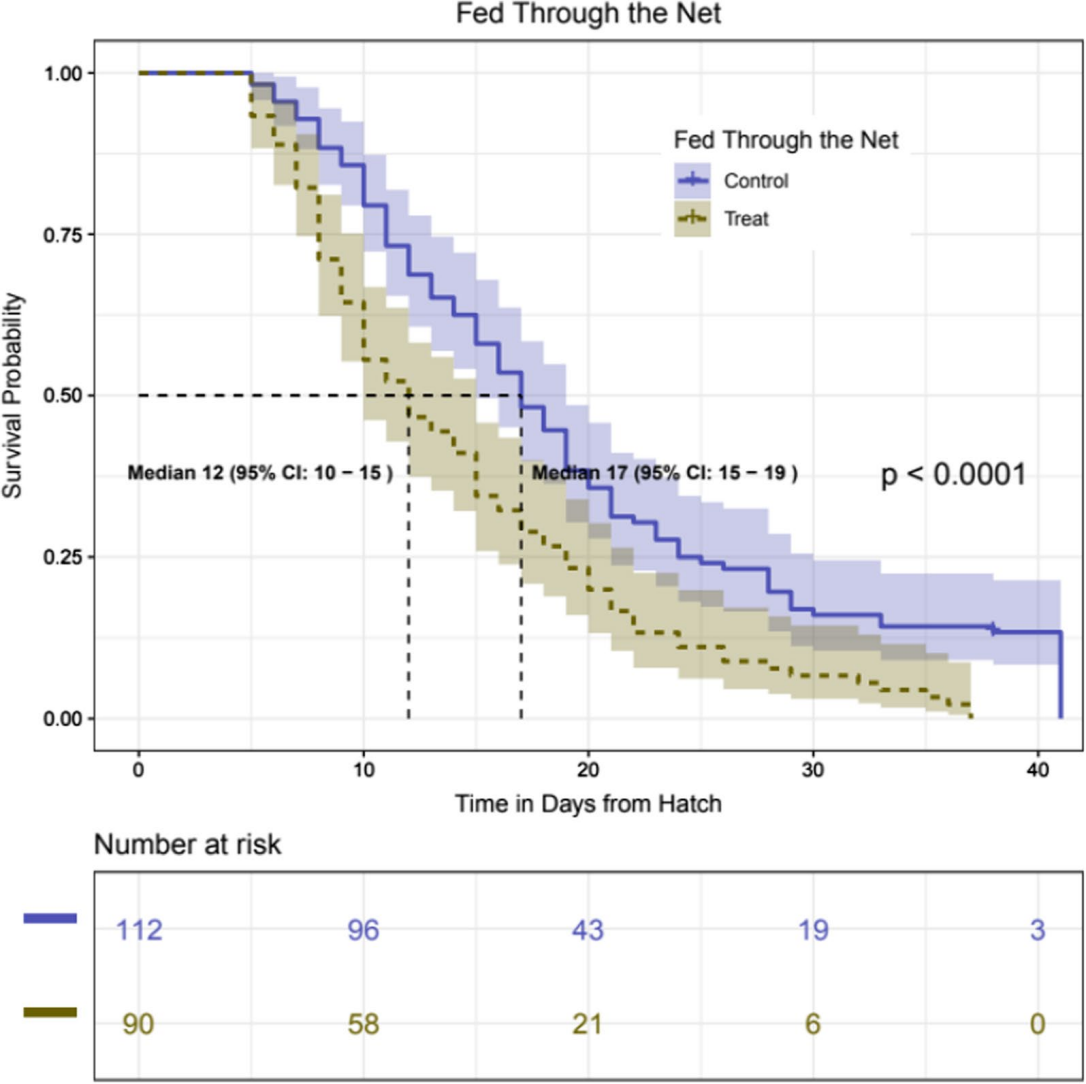
Daily survival of *An. gambiae s.s.* Kisumu strain mosquitoes after feeding on blood through a 1% PPF (brown) or an untreated net (blue). Mosquitoes were exposed to netting 4 days post-emergence

In the previous experiments, mosquitoes were exposed to a net for 3 min. To assess the impact of exposure during blood feeding, mosquitoes were presented with a blood meal for 20 min through either an untreated or PPF-treated netting. Exposure to PPF-treated netting resulted in a reduction in median adult longevity of 5 days (Fig. 3). Sterilization was not complete with this exposure regime, but there was a 5.4-fold reduction in average eggs/blood-fed female after the first blood meal, increasing to an 18-fold reduction after the second blood meal and null egg batches thereafter (Table 3). When blood-fed females were separated for oviposition, 81% (total n = 54) laid eggs in the control group compared to 29% (n = 48) in the PPF exposed group (p < 0.01).

**Table 3:** Number of blood-fed mosquitoes and number of eggs produced after different blood meals after feeding through PPF-treated and untreated netting, in mosquitoes from Kisumu strain

Netting Type	1st Blood meal		2nd Blood meal		3rd Blood meal		4th Blood meal		5th Blood meal	
	No. blood-fed / Total (%)	No. of eggs	No. blood -fed	No. of eggs	No. blood -fed	No. of eggs	No. blood-fed	No. of eggs	No. blood-fed	No. of eggs
Untreated	107 / 112 (96%)	4,218	49	4,269	43	3,022	20	1,525	17	1,514
PPF-treated	91 / 91 (100%)	654	59	287	25	0	6	0	0	0

### Survival and adult longevity are reduced by exposure to pyriproxyfen treated net in pyrethroid-resistant *Anopheles gambiae s.l.* colonies and field collected mosquitoes

Total mosquito survival 24 h post exposure was lower (p < 0.05, Fisher exact test), when field collected mosquitoes were exposed to PPF-ITNs (76% survival, n = 454) in WHO cone assays compared to standard ITNs (94% survival, n = 294) (Additional file 3: Fig. S2) although, when separated by village, the difference was only significant for mosquitoes from one of the three collection sites. Survival was high following exposure to both net types in all villages (> 89% for ITNs and > 67% for PPF-ITNs (Additional file 3: Fig.S2)). The pyrethroid resistance status of the laboratory resistant mosquito strain was confirmed by the very low mortality observed 24 h after exposure to an ITN (3%).

**Fig. 4:**
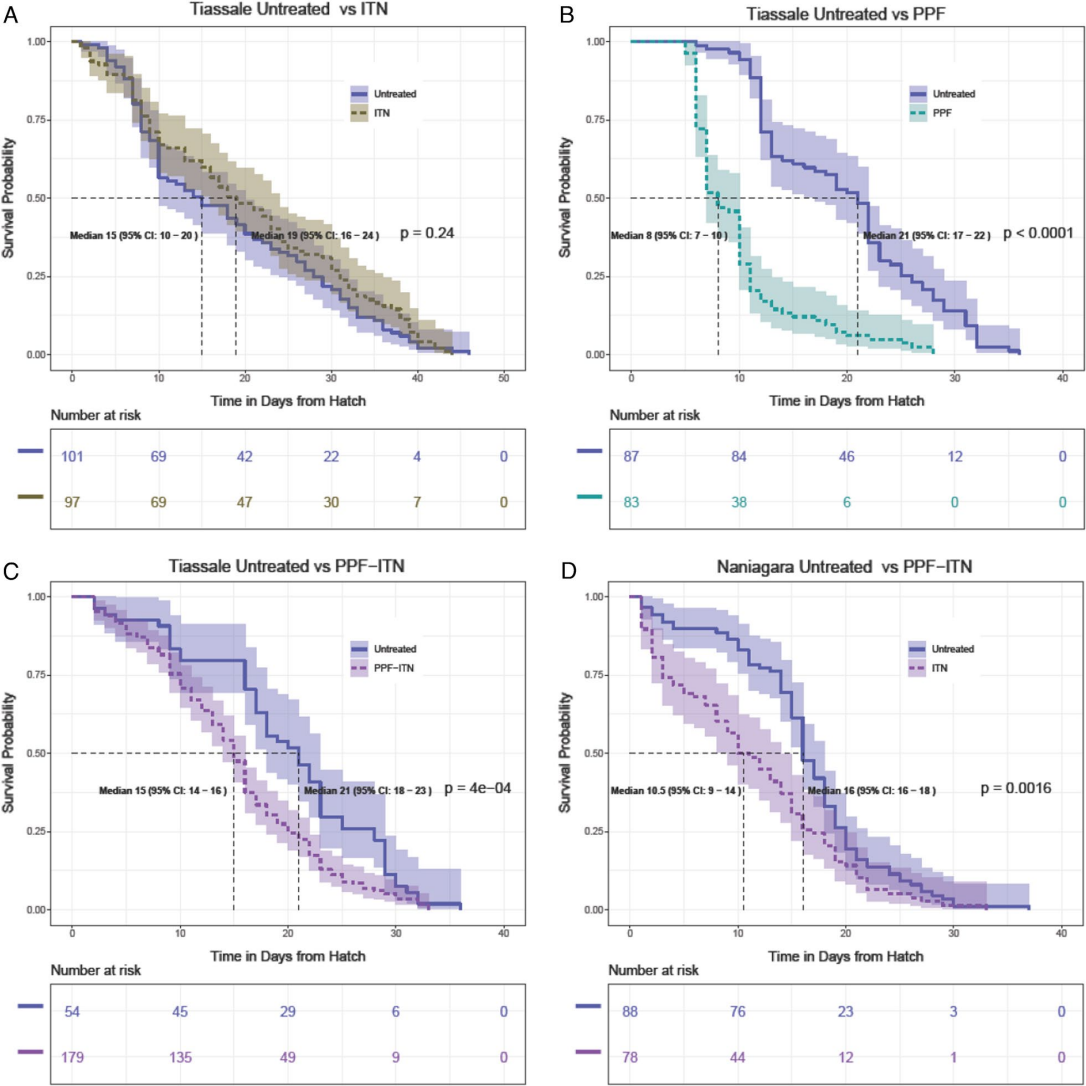
Survival curve of *Anopheles gambiae* mosquitoes from pyrethroid-resistant laboratory colony and field collected mosquitoes exposed to different treatments. **A** Pyrethroid-resistant mosquitoes exposed to permethrin-treated nets (n = 97) and untreated nets (n = 101), **B** Pyrethroid-resistant mosquitoes exposed to pyriproxyfen (PPF)-treated (n = 83) and untreated nets (n = 87), **C** pyrethroid-resistant mosquitoes exposed to PPF-ITNs (n = 179) and untreated nets (n = 54), and **D** field caught mosquitoes exposed to PPF-ITNs (n = 78) and untreated nets (n = 88). Mosquitoes were exposed to a treated or untreated net 4 days post-emergence and offered a blood meal every 7 days

The lifespan of the laboratory resistant strain was unaffected by exposure to the ITN (Fig. 4, panel A). However, the lifespan of both the resistant strain and field collected Naniagara mosquitoes was significantly reduced by exposure to a PPF-ITN (Fig. 4, panels C and D, respectively). These reductions were not just statistically but biologically significant, with median lifespan being reduced by > 5 days in PPF-ITN exposures resulting in a median lifespan in the pyrethroid-resistant laboratory strain and Naniagara field population of 15 days (95% CI 14–16) and 10.5 (95% CI 9–14) days respectively after exposure to PPF-ITNS (compared with those exposed to a control net of 21 (95% CI 18–23) and 16 days (95% CI 16–18), respectively).

### Determining the impact of implementation of Pyriproxyfen ITNs on the reproductive output of *Anopheles gambiae s.l.* in Banfora district, Burkina Faso

Blood-fed, half-gravid and gravid mosquitoes from five clusters within the Olyset Duo^®^ clinical trial site, were collected at two time points, one pre- and one post-Olyset Duo^®^ distribution. Approximately 5% of the total mosquitoes did not show any signs of oogenesis, and a further 5% did contain eggs but the morphology of the ovaries could not be clearly discerned (i.e. eggs were too small so they could be still in development, or simply no clear decision could be made upon dissections). These mosquitoes were excluded from the subsequent analyses and the remaining mosquitoes classified into three categories: 1) mosquitoes that laid eggs, 2) mosquitoes that retained normal eggs and 3) mosquitoes whose ovaries had no follicular growth or yolk deposition (defined as ‘abnormal’).

**Fig. 5:**
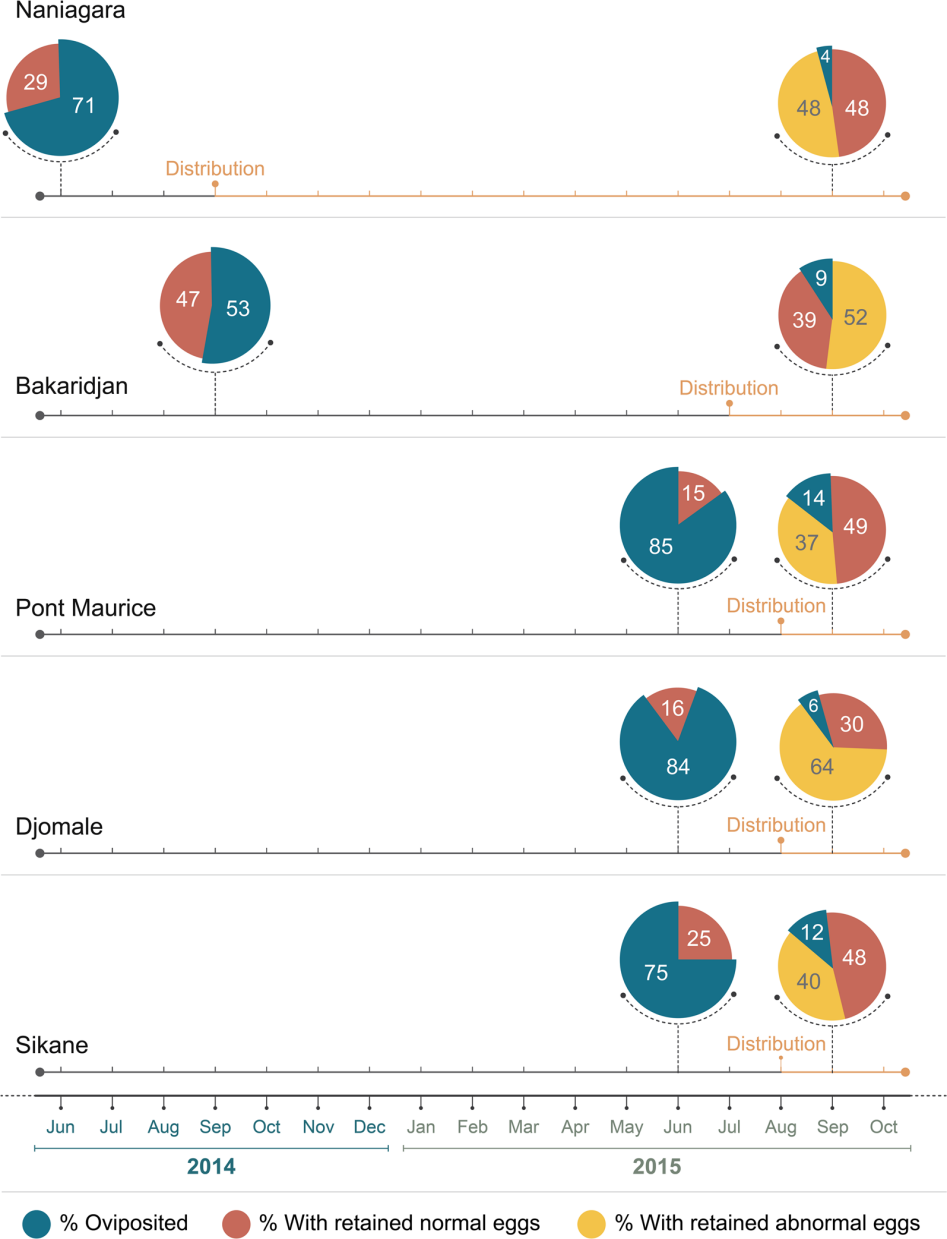
Oviposition rates and ovarian status of mosquitoes collected before and after the distribution of PPF-ITNs replaced pyrethroid only ITNs. In Naniagara (n = 72 before distribution; n = 183 after) the second round of collections was completed one year after PPF-ITNs were distributed, in Bakaridjan (n = 87 pre-distribution; n = 127 post-distribution) the second round of collections was 3 months after the change in nets and in Pont Maurice (n = 104 pre-distribution, n = 107 post-distribution), Djomale (n = 118 pre-distribution, n = 90 post-distribution) and Sikane (n = 134 pre-distribution, n = 124 post-distribution) 1 month

Of the 515 mosquitoes collected from five villages prior to Olyset Duo^®^ distribution, the proportion of female *An. gambiae s.l.* that oviposited varied by site from 53% in Bakaridjan (the only site where collections were performed in September and not June) to 85% in Pont Maurice (Fig. 5). Of these, 386 (75%) oviposited eggs normally and 129 (25%) did not oviposit but upon dissection appeared to have normal eggs. None of the non-ovipositing mosquitoes were scored as having abnormal ovaries. In contrast, of the 631 mosquitoes collected after PPF-nets were distributed only 54 (9%) successfully oviposited; 276 (44%) did not oviposit but were scored as having normal ovaries upon dissection, and 301 (48%) did not oviposit and had abnormal eggs (as in Fig. 5). Most mosquitoes collected (> 74% in each collection round) were blood-fed (Additional file 1: Table A3), and only small percentages were non-blood-fed and gravid.

**Fig. 6:**
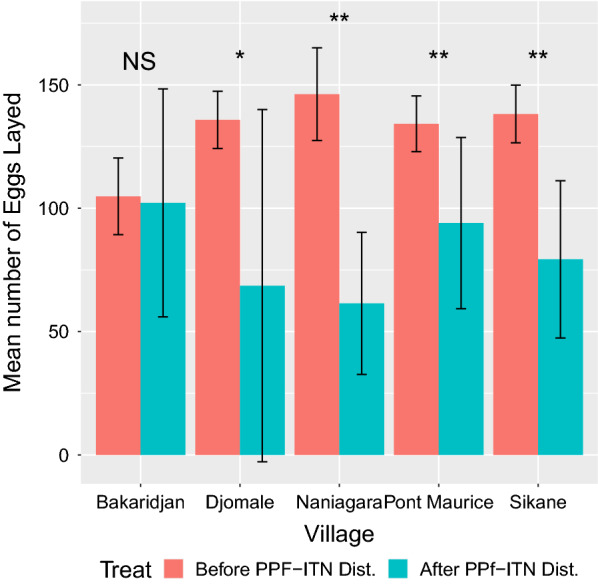
Mean number of eggs laid by Anopheles collected before and after the distribution of PPF-ITNs replaced pyrethroid only ITNs. Mosquitoes laying no eggs were removed from this analysis. The error bars show the Standard Error of Mean (SEM), and significant differences between collections within each village are p < 0.05*, and p < 0.001**

In four of the five villages surveyed, the mean number of eggs laid by each female mosquito was significantly lower in all sites in the latter collections, post distribution of the PPF-ITNs (Fig. 6). When combining data from all five villages, the overall reduction in number of eggs laid was significant (p = 0.023, paired t-test). The hatch rate of these eggs was also lower in all sites after PPF-ITN distribution (Additional file 4: Fig. S3); on average 42% of eggs laid from females collected before PPF-ITN distribution hatched vs 15% after distribution but large variations in hatch rate were observed and the difference pre and post distribution was only significant in two of the five villages.

Mean wing length was 3.18 mm (95% CI 3.16–3.21 mm, n = 190) and there was no significant association between wing length and the probability of a mosquito developing normal eggs (p = 0.197). There was also no significant difference between species composition in the subset that had normal ovary development (2 *Anopheles arabiensis*, 9 *An. coluzzii* and 54 *An. gambiae s.s.*) compared to those with abnormal ovaries (11 *An. coluzzii* and *63 An. gambiae s.s.*).

## Discussion

These findings show that PPF is a potent sterilising agent for *An. gambiae s.l.* in the laboratory and field and that PPF exposure reduces the longevity of mosquitoes.

### Impact of PPF exposure on reproductive output

Exposure to PPF-treated nets in the laboratory completely prevented egg laying when mosquitoes were exposed 24 h prior to blood feeding or 24 h post blood meal, and this sterilising effect was retained after five successive rounds of weekly blood feeding. PPF exposure closer to the time of blood feeding (6 h or immediately prior) also resulted in complete loss of oviposition for the 1st gonotrophic cycle, with only a very small number of eggs laid (maximum average of 1.4 eggs/blood-fed female) following subsequent blood meals. The decline in egg production was not due to a reduction in blood feeding and is indicative of a direct impact of PPF on egg development. In mosquitoes exposed to PPF, those that did not oviposit failed to develop morphologically normal eggs as seen previously [26, 27]. The mechanism by which PPF achieves lifelong sterilization may be due to permanent disruptions in JH-mediated gene regulation [4], absence of nurse cell degeneration in follicle development [28], lack of follicle reabsorption [28], irreversible damage of the reproductive organs [8] or a combination of these.

When mosquitoes were exposed to PPF during a blood meal, sterilization was partial, but the total number of eggs per original blood-fed mosquito over 5 successive blood meals reduced by > 13-fold from 136 in mosquitoes exposed to untreated netting to 10.3 in those exposed to PPF netting. In this experiment, the length of exposure was not controlled as mosquitoes were allowed to feed naturally until replete. Reduced impact of exposure to pyrethroids in groups who also received a blood meal has been observed in laboratory trials [29, 30] and in experimental hut trials [31], possibly due to the upregulation of detoxification enzymes during digestion of a blood meal providing some protective effect [32, 33]. It is possible that a similar phenomenon is being observed here.

Lifelong, almost complete sterilization, occured after PPF exposure, and is consistent with previous studies of insecticide susceptible *An. gambiae* exposed to concentrations of PPF that were orders of magnitude lower than the 1% PPF on the nets in this study [8]. Mbare and co-workers [34] also recorded a strong sterilising effect on *An. gambiae s.s.* females exposed within 24 h before or after a blood meal. However others found that sterilization in *An. arabiensis* only occurred in mosquitoes exposed to PPF 24 h after blood feeding, but not 24 h before [35]. Jaffer [36] found that greatest fertility inhibition was induced in *An. gambiae* when females were blood-fed 1 h after exposure, compared to 24 and 120 h after, concluding that the effects of pyriproxyfen are partially reversible through the action of metabolism or excretion. The differences observed on the impact of timing of exposure on sterilization effect between the studies could have multiple explanations including differences in species or strain sensitivity, duration of exposure, the formulation and concentration of pyriproxyfen, the surface mosquitoes were exposed to and the specific nature of the bioassay. However, these laboratory results that showed substantial sterilization regardless of the timing of exposure were encouraging as under operational settings, female mosquitoes may encounter active ingredients on bed nets whilst host seeking (i.e. prior to blood feeding), during the act of feeding, or whilst trying to escape a holed net after successfully blood feeding.

To the best of the authors’ knowledge, this study is only the second study to measure the impact of PPF-ITNs on mosquito reproduction under field conditions. The earlier study was a small-scale study involving collections in 15 households which found an overall reduction in the proportion of blood-fed mosquitoes ovipositing one week after the introduction of PPF-treated nets from 77 to 45% with a nearly 50% reduction in number of eggs/female [9]. The current study was considerably larger than the previous study and involved collections from 286 households using PPF-treated nets. Importantly, from the results in Naniagara we were able to show the sterilization persisted for one year after the PPF-treated nets were deployed in the field. In these experiments the average number of eggs laid by female *An. gambiae s.l.* and the egg hatching rate declined after deployment of PPF-treated nets compared with the period before deployment when standard pyrethroid-treated nets were used. This effect was not related to the size of the mosquitoes or species composition at the different collection time points. The absence of abnormal ovaries before deployment of PPF-treated nets is striking.

### Impact of PPF exposure on adult longevity

In laboratory assays, PPF exposure resulted in significantly reduced median lifespan of 2–5 days in all but one treatment, in agreement with earlier studies [e.g. 8]. A similar life shortening effect of between 5.5 and 7 days was observed after pyrethroid resistant populations were exposed to the mixture of permethrin and PPF. Similar findings were found with unfed mosquitoes exposed to PPF-treated netting (data not shown). Whilst exposure to pyrethroid only nets has previously been shown to reduce adult longevity [37], in the current study exposure to pyrethroid-treated ITNs had no effect on longevity, strongly suggesting that the reduction in lifespan seen from exposure to PPF-ITNs is caused by PPF. In order for a mosquito to transmit malaria parasites they must survive the 10–14 day intrinsic incubation period for the parasite to develop between first and subsequent blood meals. Hence if the reductions in lifespan observed in the laboratory were indicative of a similar effect in the field, nets containing PPF would be expected to lead to major reductions in malaria transmission by reducing the proportion of infected mosquitoes. This is supported by a decline in parous mosquitoes observed in villages with PPF-treated nets (Odds ratio = 0.69 (0.52–0.91)) in a cluster randomized controlled trial in Burkina Faso [17]. Mathematical modelling shows that a decrease in mosquito longevity would have a greater impact on transmission than sterilization [39]. It is important, however, to note that daily mosquito survival in the field is much lower than observed in the laboratory. In nature few females survive long enough to become infectious [40], even in the absence of PPF. Thus the impact of PPF-treated netting in the field is likely to be greater than indicated by our laboratory experiments.

### Other impacts of PPF exposure

Previous studies have shown that the mortality of mosquitoes exposed to PPF-ITN netting was greater than when exposed to pyrethroid only treated nets and a similar result was observed in our study when results were pooled for the three villages. Although the concentration of permethrin in the standard ITNs and PPF-treated ITN were identical, the bleed rate in the PPF-treated ITNs was higher than the standard net, suggesting that there was likely to be higher concentrations of permethrin on the PPF-treated ITNs than the standard net. Alternatively, the increased mortality observed when permethrin is combined with PPF may be due to competitive metabolism delaying the detoxification of the pyrethroid insecticide; this hypothesis is supported by studies showing that certain mosquito P450s can efficiently metabolize both permethrin and PPF [42]. Although this study did not measure P450 gene expression levels in the field collections from individual villages, it has subsequently been found that CYP6M2, CYP6P3 and CYP6P4, all confirmed metabolizers of both PPF and pyrethroids, are over expressed in multiple populations of *An gambiae s.l.* from the Cascades region of Burkina Faso (Ranson and N’Falé, unpublished data).

In addition to incorporation into bed nets, PPF is already used to suppress mosquito populations either by directly applying to breeding sites, or by adding PPF powder to odour baited traps. Both approaches can effectively reduce adult emergence and the latter has the added advantage of using the mosquito itself to autodisseminate PPF to a wide range of breeding sites, including cryptic sites difficult to reach with conventional larvicides [43]. There was no direct test for autodissemination of PPF by mosquitoes exposed to the PPF nets as the chemical steps needed to incorporate PPF (or any active ingredient) into the net are intended to release the chemical in a slow controlled manner and it is anticipated that the formulated product would not be as readily disseminated as the powdered PPF used in traps. The effect of PPF exposure on the development of *Plasmodium* in the mosquito was not investigated; this should be the subject of further studies given the impact that agonists of the steroid hormone 20-E have been shown to have on prevalence of establishment of *Plasmodium* infections in the mosquito midgut [39].

### Implications for use of PPF in insecticide treated nets

The results of this study show that PPF is highly effective in reducing reproductive outputs and adult longevity of both insecticide susceptible and pyrethroid resistant mosquitoes. A clinical trial in the study area showed that the addition of PPFs to ITNs can reduce the clinical burden of malaria in an area of high intensity transmission by 12%. This reduction in malaria was associated with fewer vectors and a decrease in the survival of the vector population, leading to a halving in the entomological inoculation rate (EIR) in villages with PPF-treated nets compared to those with standard ITNs. The observation of reduced adult female density in clusters of villages with PPF-ITNs suggests that the reduction in the number of immature *Anopheles* caused by the impact of PPF on mosquito fertility was not fully compensated for by any increase in larval productivity in the natural aquatic habitats caused by reduced larval densities. Whether a similar relationship between female fertility and adult population size would be observed in other transmission settings remains to be seen. Linking the entomological effects of PPF exposure with epidemiological outcomes is the subject of ongoing studies and may inform future development of nets containing insect growth regulators. Laboratory tests of an agonist of the steroid hormone 20-ecdysone [39] recorded four distinct effects (reduced insemination, reduced egg production, shortened lifespan and impaired *Plasmodium* development) not all of which have been investigated for PPF. Determining the importance of each potential entomological outcome on the epidemiological impact of nets containing PPF and pyrethroids under field settings, with different species compositions and differing levels of pyrethroid resistance, is important for establishing where and when this potential new net class may be most effective.

In the current study, evidence is provided that PPF-ITNs remained effective in reducing *Anopheles gambiae* reproductive output for up to a year after distribution. However, a net durability study on Olyset Duo^®^, performed alongside the clinical trial found that impacts on mosquito fertility were lost after one month of operational use (although the increase in 24 h mortality in PPF-ITNs compared to standard ITNs was retained for the duration of the study) [41]. The disparities between the two studies may reflect differences in the bioassays deployed; understanding the correlation between entomological outcomes may help guide the development of simple robust protocols to measure PPF bioefficacy on nets. It should also be noted that these results refer to specific net formulations. Other nets containing PPF will have differing release profiles and so will perform differently, meaning that additional studies would be needed in order to evaluate their efficacy.

There are a number of limitations to our study. Firstly, the evaluation of the efficacy of PPF-ITNs in reducing reproductive output was a pilot study; available resources did not permit collection of mosquitoes from additional replicate villages at each time interval, which would have supported more robust conclusions. Secondly, we did not include a further follow up beyond 12 months to test whether the sterilization effect lasted for the full expected lifespan of the net. Thirdly, although mosquitoes were only sampled from houses containing PPF-ITNs at the ‘post distribution’ time point, there is no way of knowing whether these mosquitoes actually came into contact with the nets prior to sampling. Fourthly, bioassay methods, mosquito strains and blood sources varied across the different arms of the study, potentially confounding interpretation of the results. Finally, the lack of reliable methods to age adult mosquitoes meant it couldn’t be established whether the reduction in longevity following PPF-ITN exposure observed in the laboratory was replicated under operational conditions.

## Conclusions

The mixture of PPF and pyrethroids in a net can increase contact mortality, reduce reproductive outputs and shorten the lifespan of pyrethroid-resistant mosquitoes. In laboratory colonies of *An. gambiae s.l.* these effects are largely independent of the time of exposure relative to a blood meal. Importantly the PPF in PPF-ITNs remained active on the nets for at least a year under operational settings; this contrasts with results from laboratory cone bioassays which found the sterilising effect of PPF was lost after one month of net use and highlights the importance of developing and utilising assays that are reflective of the performance of products in the field. The data presented will guide the development of laboratory assays to assess efficacy and durability of different products and provide encouragement that the public health value of this potential new net class may be realized in future clinical trials.

## Supplementary Information

**Additional file 1: Table A1.**Geographic information of the villages where mosquitoes were collected for the study. ** Table A2**: Details of mosquito strains used in each experiment.**Table A3.**. Physiological status of the female Anopheles collected pre and post PPF-ITN deployment.

**Additional file 2: Figure S1.** Deli pot bioassay.

**Additional file 3: Figure S2.** Susceptibility of field mosquitoes to ITNs and PPF-ITNs.

**Additional file 4: Figure S3.** Hatch rate of eggs laid by Anopheles collected before and after the distribution of PPF-ITNs replaced pyrethroid only ITNs.

## Data Availability

The datasets generated and/or analysed during the current study are available from the corresponding author on reasonable request.

## Abbreviations

EIR: Entomological inoculation rate
ITN: Insecticide treated net
LSTM: Liverpool School of Tropical Medicine
ND: Not determined
PPF: Pyriproxyfen
WHO: World Health Organization
