# Supplementary material for: Pyriproxyfen-treated bed nets reduce reproductive fitness and longevity of pyrethroid-resistant Anopheles gambiae under laboratory and field conditions
Source: Malar J. 2021 Jun 22;20:273. doi: 10.1186/s12936-021-03794-z (PMC8218427; doi:10.1186/s12936-021-03794-z)
Supplement: Supplementary file 1 — Additional file 1: Table A1.Geographic information of the villages where mosquitoes were collected for the study. Table A2: Details of mosquito strains used in each experiment.Table A3.. Physiological status of the female Anopheles collected pre and post PPF-ITN deployment. [file 12936_2021_3794_MOESM1_ESM.docx]

## Additional Files

Supplementary Table A1**.** Geographic information of the villages where mosquitoes were collected for the study.

| Health Centre | Village | Coordinates |
| --- | --- | --- |
| Tiefora | Djomale | 10˚33’17.24’’N; 4˚22’41.14’’W |
|  | Pont Maurice | 10˚38’26.71’’N; 4˚29’41.62’’W |
|  | Sikane | 10˚34’27.49’’N; 4˚22’38.16’’W |
|  |  |  |
| Kankounadeni | Naniagara II | 10˚32’9.15’’N; 4˚40’7.84’’W |
|  |  |  |
| Koflande | Bakaridjan II | 10˚24’26.34’’N; 4˚33’44.78’’W |

Supplementary Table A2: Details of mosquito strains used in each experiment

| Experiment | Mosquito strain | Characteristics of the mosquito strain 2015 (permethrin discriminating doce bioassy % mortality) |
| --- | --- | --- |
| Effect of relative time of exposure to pyriproxyfen and blood feeding: longevity and lifelong fecundity | Kisumu, Kenya 1975 | Laboratory colony, susceptible to pyrethroids and other insecticides (100 %) |
| Effect of relative time of exposure to pyriproxyfen and blood feeding: fecundity, fertility and offspring viability | Kisumu | Laboratory colony, susceptible to pyrethroids and other insecticides |
| 5) | Tiassalé 13, Cote d’Ivoire, 2013 | Laboratory colony, highly resistant to pyrethroids, DDT , dieldrin, bendiocarb and propoxur. (0 %) |
|  | Naniagara  Tiefora  Bakaridjan | Field collected mosquito populations;  resistant to pyrethroids  Naniagara (20 %; 95 % CI 13-24%)  Tiefora (1 %; 95 % CI 0-6)  Bakaridjan (17 %; 95 % CI 11-24 %) |
| Measurement of individual mosquito fecundity, fertility and offspring viability of wild mosquitoes | Naniagara  Bakaridjan  Pont Maurice  Djomale  Sikané | Field collected mosquito populations;  resistant to pyrethroids  Pont Maurice (18 % ; 95 % CI 11-29%)  Djomale (14 % 95 % CI 8-23 %)  Sikané (22 %; 95 % CI 14-31 %) |

## Supplementary Table A3. Parity pre- and post-deployment. Physiological status of the female Anopheles. Total percentages of blood-fed, gravid and non-blood-fed mosquitoes collected before and after the distribution of Olyset Duo®. The ‘blood-fed’ entry includes the mosquitoes that had that status but died upon collection and the mosquitoes that were used in the oviposition assays (Figures 6, 7 and 8).

| **Village** | **Intervention** | **n** | **Blood-fed** | **Gravid*** | **Non-blood-fed*** |
| --- | --- | --- | --- | --- | --- |
| Naniagara | Before Duo | 92 | ND | ND | ND |
|  | After Duo | 291 | 83.2 % | 7.6 % | 9.3 % |
|  |  |  |  |  |  |
| Bakaridjan | Before Duo | 202 | ND | ND | ND |
|  | After Duo | 184 | 84.8 % | 11.4 % | 3.8 % |
|  |  |  |  |  |  |
| Pont Maurice | Before Duo | 213 | 99.1 % | 0 | 0.9 % |
|  | After Duo | 220 | 74.5 % | 5.9 % | 19.5 % |
|  |  |  |  |  |  |
| Djomale | Before Duo | 361 | 94.5 % | 0 | 5.5 % |
|  | After Duo | 117 | 80.3 % | 5.1 % | 14.5 % |
|  |  |  |  |  |  |
| Sikane | Before Duo | 239 | 98.7 % | 0 | 1.3 % |
|  | After Duo | 195 | 94.4 % | 1.5 % | 4.1 % |

*Dead on the day of collection

*Supplementary Figure S1:* **Deli pot bioassay**. A) Components needed for the bioassay; B) Deli pot assembled and ready for the bioassay; C) Mosquito introduction inside the plastic pot, using a standard aspirator and a piece of Parafilm to close the orifice; D) Mosquitoes in contact with the target net.

Supplementary Figure 2: **Susceptibility of field mosquitoes to ITNs and PPF-ITNs.** F_0_ progeny from larvae collected from Tiefora, Naniagara and Bakaridjan were exposed to and ITN (Olyset Net) and Olyset Duo in a WHO cone test and scored for mortality 24 hours post exposure. Error bars represent 95% confidence intervals. Z-tests significant differences: *** = p<0.001. The red line represents the minimum effectiveness of ITNs recommended by WHO (WHO, 2013). Numbers over each bar represent the number of mosquitoes tested per net.

Supplementary Figure 3: **Hatch rate of eggs laid by Anopheles collected before and after the distribution of PPF-ITNs replaced pyrethroid only ITNs.** The hatch rate was measured as the percentage of eggs that reached second instar larvae over the total number of eggs laid. Error bars: 95% CI. The P value for difference of proportion of oviposited eggs that hatched, before and after net distribution. Significant differences between collections within each village are shown over each bar.
