# Supplementary figures and images for: Pyriproxyfen-treated bed nets reduce reproductive fitness and longevity of pyrethroid-resistant Anopheles gambiae under laboratory and field conditions
Source: Malar J. 2021 Jun 22;20:273. doi: 10.1186/s12936-021-03794-z (PMC8218427; doi:10.1186/s12936-021-03794-z)

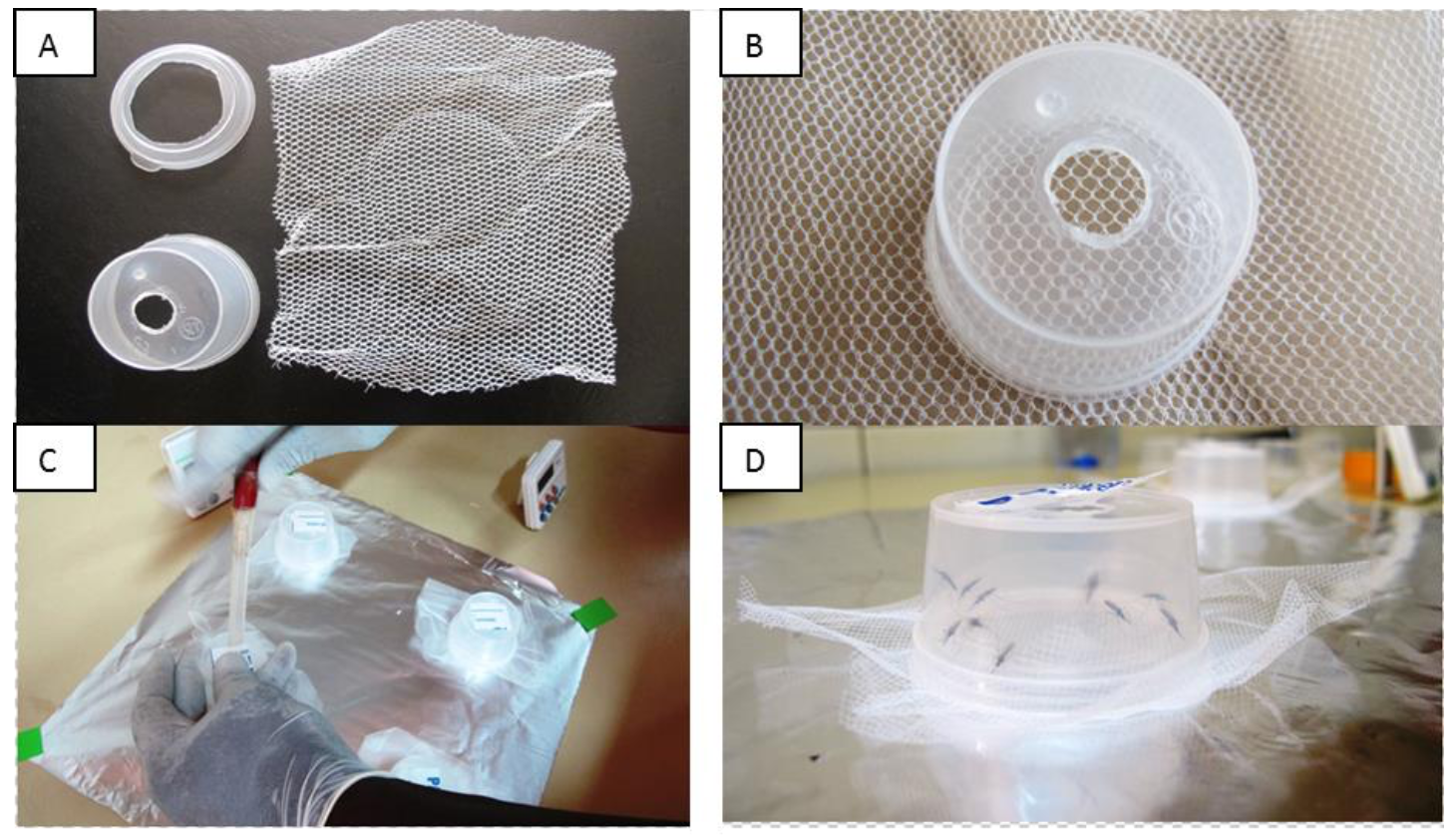
**Figure S1. Deli pot bioassay**.

Supplement: Supplementary file 2 — Additional file 2: Figure S1. Deli pot bioassay. [file 12936_2021_3794_MOESM2_ESM.docx]

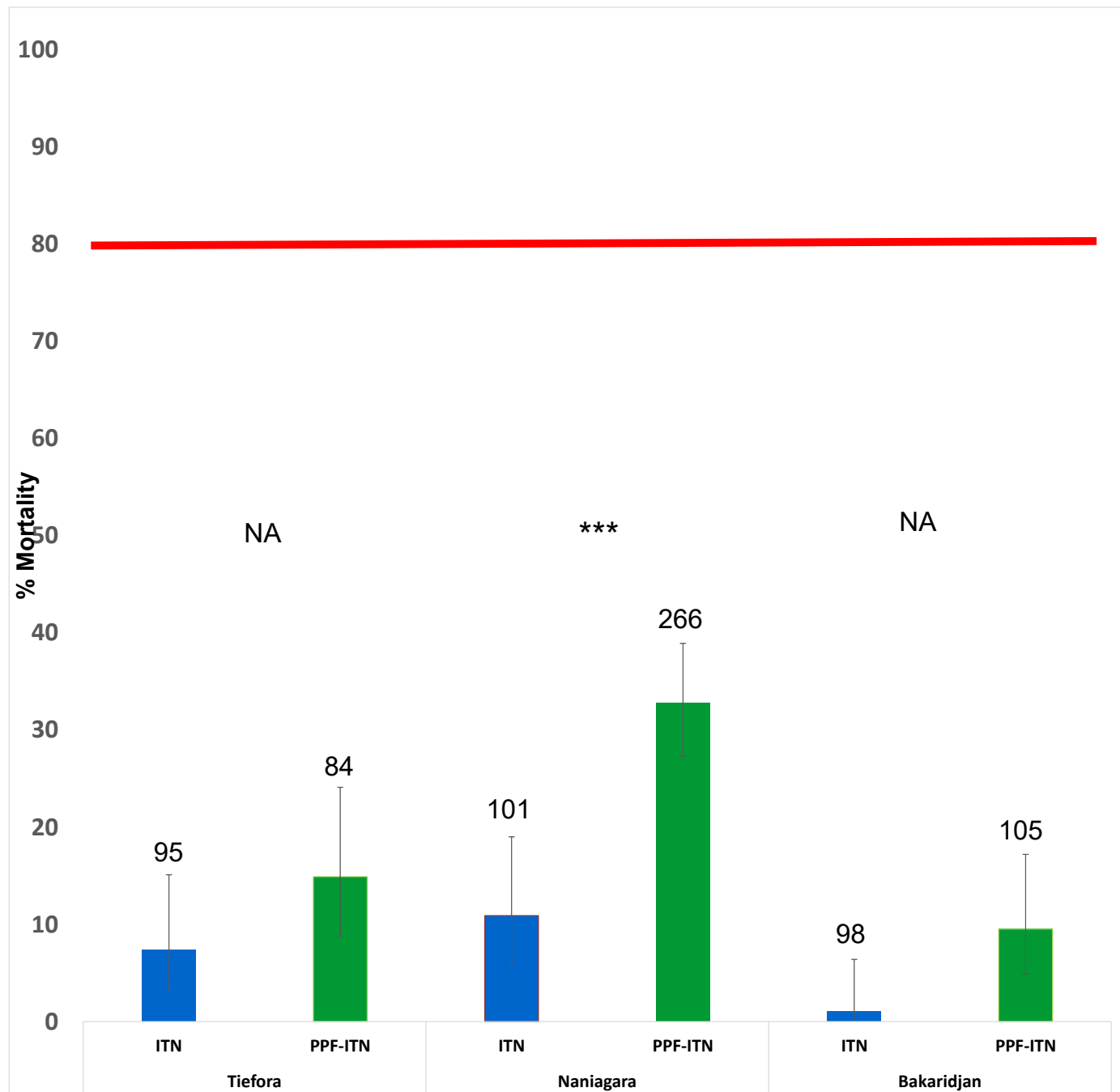

Supplement: Supplementary file 3 — Additional file 3: Figure S2. Susceptibility of field mosquitoes to ITNs and PPF-ITNs. [file 12936_2021_3794_MOESM3_ESM.pdf]

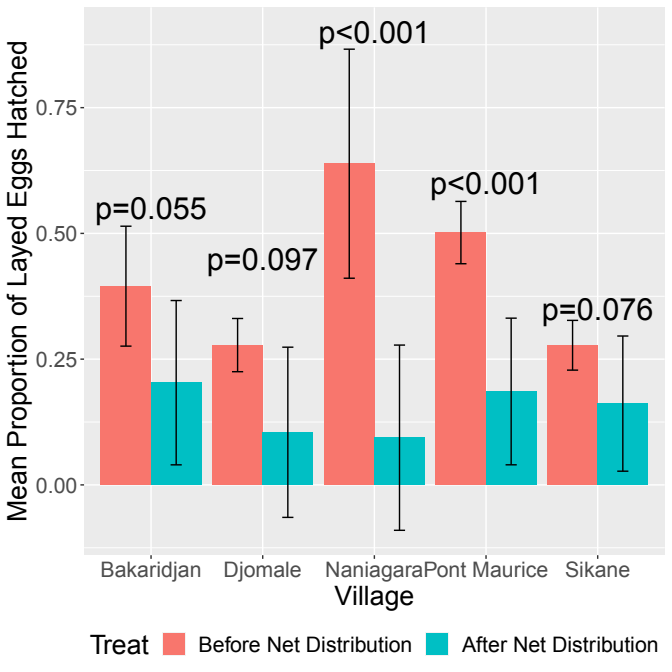

Supplement: Supplementary file 4 — Additional file 4: Figure S3. Hatch rate of eggs laid by Anopheles collected before and after the distribution of PPF-ITNs replaced pyrethroid only ITNs. [file 12936_2021_3794_MOESM4_ESM.docx]
